# National survey on availability, use and clinical impact of point-of-care blood analysis systems in Swedish emergency departments

**DOI:** 10.1186/s12873-025-01251-7

**Published:** 2025-06-07

**Authors:** Matthias Jörg, Tim Lundgren, Jens Wretborn, Daniel B. Wilhelms

**Affiliations:** 1 Department of Emergency Medicine, Sundsvall Regional Hospital, Region Västernorrland, Sweden; 2 Department of Biomedical and Clinical Sciences, Linköping University, Linköping, 58185 Sweden; 3 Faculty of Medicine and Health Sciences, Linköping University, Linköping, 581 83 Sweden; 4 Department of Emergency Medicine, Local Health Care Services in Central Östergötland, Region Östergötland, Sweden

**Keywords:** Blood gas, POCT, Analysis, Usage, Workflow, Reliability, Cost-effectiveness, Emergency department

## Abstract

**Background:**

Expedited diagnostic results are important in time sensitive operations like the Emergency Department (ED). Point-of-care testing (POCT) is used for a broad range of blood tests reducing wait times and providing faster decisions on patient management compared to central laboratory analysis. If POCT is to substitute central laboratory analysis as part of the decision making process in the ED, cognizance on the distribution and availability of the required instruments, staff training and workflow integration is essential. The aim of this study was to determine the accessibility of POCT, with particular emphasis on blood gas analysis, in all Swedish EDs and to describe its incorporation into workflow.

**Methods:**

This was an exploratory survey study conducted in 2024 using a digital questionnaire focusing on POCT availability, perceived reliability, sampling, analysis and workflow integration. Descriptive data is reported as percentage, mean with standard deviation. Qualitative data in open-ended questions was analyzed in a descriptive manner and systematically summarized.

**Results:**

All Swedish EDs (*n* = 71) responded to our survey. All EDs utilized some kind of POCT with the most prevalent being blood gas, Hb, glucose and CRP. 75% of all EDs had at least one blood gas instrument on site. 44% had guidelines to define indication and usage, 67% replaced certain central laboratory analyses in favor of POCT. ED staff assessed the reliability of results of POCT blood gas analysis as 8 on a 1–10 likert scale (SD 1). All EDs provided staff training on sampling and analysis, but only 30% of EDs had repeated training sessions to avoid misoperation and preanalytical errors. In 32% of reporting EDs, POCT blood gas analysis was performed on more than 30% of patients, mainly triggered by standard operating procedures.

**Conclusion:**

All Swedish EDs utilized basic POCT to accelerate patient flow. The majority of Swedish EDs have access to POCT blood gas analysis, the replacement of central laboratory analyses was common. Perceived reliability of POCT blood gas analysis results was considered high, despite the fact that staff training varied substantially. The overall utilization of other POCT remains low – that despite good evidence for cost-effectiveness, reliability and shortened length of stay.

**Trial registration:**

Clinical trial number: Not applicable.

**Supplementary Information:**

The online version contains supplementary material available at 10.1186/s12873-025-01251-7.

## Introduction

### Background

Expedited diagnostic results are important in time sensitive operations, like the Emergency Department (ED) or intensive care unit (ICU) and reducing wait times for diagnostic tools such as blood tests and imaging can provide faster decisions on management and patient disposition [1]. This has led to the development of point-of-care testing (POCT) where a broad range of blood tests are sampled and analyzed within a department, in contrast to sending them to a central laboratory [2].

POCT has been shown to significantly reduce turnaround time for test results and length of stay in the ED and ICU [3, 4] with reductions of 21 min for blood gas analysis and up to 2 h and 25 min for d-dimer [5, 6]. A large multicenter study looking at POCT for cardiac markers found that four out of six hospitals successfully reduced length of stay for patients admitted to the ED with chest pain [7]. Additionally, several studies have found a consistent reduction in time to management decisions and reduced wait times for diagnostic imaging with POCT creatinine results [1, 5, 8]. POCT troponin improved 30-day readmission rates and in-hospital death rates by correct initial patient triage and transport prioritization for definitive care in patients in rural areas with suspected heart disease [9]. Recently, POCT has been included as a central part in ED triage systems: The Swedish West coast System for Triage (WEST), based on the South African Triage Scale (SATS) [10], presupposes POCT venous blood gas for the initial assessment of the acutely ill patient to speed up management decisions [11]. Altogether, POCT is becoming an integral part in many aspects of ED operations.

POCT results are more prone to divergent physiological states e.g. lipidemia, icterus and hemolysis compared to central laboratory testing [12]. Hemolysis, which may increase potassium, was found to be a potent confounder in multiple studies leading to changes in management decisions and potential patient harm [12–14]. However, other studies show contrary results with a good correlation advocating for a broader use of POCT [15–17]. If POCT is to substitute central laboratory analysis as part of the decision making process in the ED, reliability is essential. One study has shown that ED physicians are more satisfied with POCT compared to routine testing in a central laboratory, but perceived reliability of POCT is unknown [18].

POCT is becoming an integral part in the assessment of the critically ill patient leveraging the ability to make appropriate clinical decisions in a timely manner. However, for broad uptake and ability to incorporate into national guidelines, availability of the required apparatus and analysers is fundamental. In Sweden, the distribution, access and extent of clinical use of POCT in EDs is unknown. To utilize the reduction in turnaround time into clinical practice, the results need to be reliable to the decision makers.

## Methods

### Aims

The aim of this study was to determine the accessibility of POCT, with particular emphasis on blood gas analysis, in all Swedish EDs and to describe its incorporation into the workflow.

### Study design and setting

This was a survey study aimed at describing POCT availability and incorporation into the workflow in Swedish EDs in 2024. All investigated EDs are hospital-based, ranging from rural hospitals to tertiary care centers.

### Data collection and participants

The data collection was carried out between October 2023 and August 2024. Study information, including a link to an online questionnaire, was sent out via email to the medical directors of all Swedish EDs. For non responders, telephone calls were made until contact with the responsible person on site was established.

The survey consisted of 24 questions in 11 sections, and data was collected via the online survey tool Webropol Survey & Reporting [19]. Closed-ended questions were used to assess whether EDs had access to different POCT methods or not, and if the instrument utilized automated transfer of the results to the patient’s health record. Further, we asked open-ended questions regarding: analysis instrument details, routines for blood gas sampling, routines for analysis of blood gas samples, routines for maintenance of the analysis instrument, how POCT blood gas analysis was incorporated into the workflow of the ED, if economical aspects are considered in the use of POCT, amount of blood gas analysis carried out per year, and finally the ED census. Likert scale questions were used to determine perceived reliability of the POCT blood gas results. The survey was conducted in Swedish and the questions are presented in full in Appendix 1.

### Statistical and content analysis

Descriptive data is reported as percentage, mean with standard deviation (SD) or median with interquartile ranges (IQR) as appropriate. Non-responder rates are shown where appropriate. Statistical analysis was performed with R (version 4.1.3) and graphs were rendered with the ggplot2 package (version 3.4.1). Qualitative data in open-ended questions was analyzed in a descriptive manner and systematically summarized.

### Ethical considerations

#### Ethical approval

was waived by the Swedish Ethical Review Board (decision number EPM 2023-02780-01). Accordingly, no consent to participate was needed and no personal information was collected for this study. Contact details to the medical directors of Swedish EDs is public information openly available from the local healthcare organizations. No patient data was collected. Clinical trial number: not applicable.

## Results

All Swedish EDs (*n* = 71) reported data (response rate = 100%). Mean census was 38,275 patient visits/year (min 4000, max 90000, SD 21368) and 33 (47%) departments reported additional data on the amount of blood gasses analyzed (Table 1).

**Table 1:** List of participating EDs in Sweden, *n* = 71

ED name	Patient visits / year, *n*	Proportion POCT blood gas of total patient visits, %	ED name	Patient visits / year, *n*	Proportion POCT blood gas of total patient visits, %
Alingsås	22,000	27	Mora	28,500	n/a
Arvika	15,000	n/a	Motala	25,000	14
Avesta	20,000	n/a	Norrköping	50,000	n/a
Ängelholm	14,100	n/a	Norrtälje	30,000	n/a
Bollnäs	17,000	n/a	Nyköping	31,000	n/a
Borås	60,000	40	Oskarshamn	18,000	n/a
Eksjö	26,000	n/a	Örebro	75,000	80
Enköping	23,000	n/a	Örnsköldsvik	22,000	n/a
Eskilstuna	54,000	80	Östersund	36,000	28
Falun	56,000	43	Piteå	16,000	n/a
Gällivare	10,400	n/a	Skaraborg	70,000	10
Gävle	43,800	14	Skellefteå	32,000	11
Gbg DSB pediatric	62,000	n/a	Sollefteå	12,800	75
Gbg Mölndal	46,500	n/a	Sthlm ALB pediatric	60,000	40
Gbg Sahlgrenska	60,000	n/a	Sthlm Danderyd	90,000	20
Gbg Östra	55,000	22	Sthlm Huddinge	55,000	68
Halmstad	45,000	n/a	Sthlm Hud pediatric	18,000	n/a
Helsingborg	75,000	64	Sthlm Solna	15,000	80
Hudiksvall	28,000	11	Sthlm St Göran	89,000	16
Hässleholm	9000	n/a	Sthlm SÖS	86,000	42
Jönköping	46,000	22	Sunderby	34,000	59
Kalix	11,900	n/a	Sundsvall	46,000	49
Kalmar	42,000	0.3	Södertälje	32,000	n/a
Karlskoga	24,000	n/a	Torsby	14,000	n/a
Karlskrona	36,000	10	Trelleborg	14,000	n/a
Karlstad	51,100	8	Trollhättan	66,500	46
Kiruna	41,000	n/a	Umeå	44,000	n/a
Kristianstad	45,000	53	Uppsala	50,000	43
Kullbergska	23,000	52	Varberg	45,000	n/a
Kungälv	32,000	n/a	Visby	27,000	n/a
Landskrona	4000	n/a	Värnamo	22,800	n/a
Lindesberg	15,200	n/a	Västerås	57,000	42
Linköping	52,000	23	Västervik	22,000	n/a
Lund	64,000	n/a	Växjö	38,000	6
Lycksele	11,000	n/a	Ystad	27,000	n/a
Malmö	78,900	n/a			

All Swedish EDs (71/71) use some kind of POCT other than blood gas analysis. Most prevalent were glucose, C-reactive protein and hemoglobin (Table 2). POCT troponin analysis is used in two EDs.

**Table 2:** Non-blood gas point-of-care blood tests available in all EDs

Point of care test	Amount (out of 71)	Percent (out of 71)
C-Reactive protein (CRP)	54	77%
Glucose	58	82%
Hemoglobin	53	75%
Ketones	34	47%
Leucocytes	9	13%
Troponin	2	3%

### Point of care blood gas analysis

75% (53/71) of all EDs had at least one blood gas instrument on site for immediate bedside use. Most EDs used Radiometer model ABL-90 (22/53, 42%) or ABL-800 flex (19/53, 36%), followed by Siemens RapidPoint 500e (9/53, 17%), Abbott i-STAT (4/53, 8%) and Werfen GEM premier 5000 (2/53, 4%).

#### Reliability

**Fig. 1:**
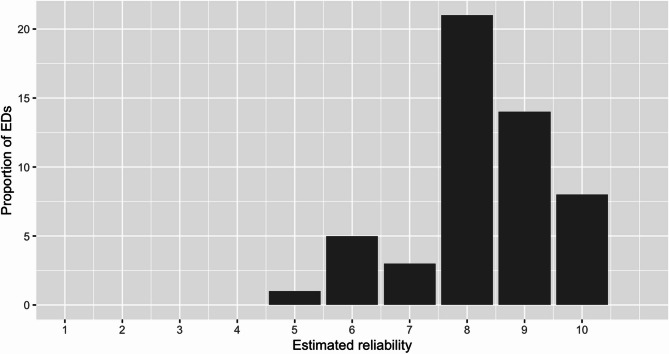
Estimation of reliability on a likert scale of 1–10 where 1 was “Not at all reliable” and 10 “Fully reliable”. x-axis showing assessment scoring, y-axis showing N of participating EDs, total *n* = 71, mean estimation 8 (± 1)

ED staff assessed the reliability of results of POCT blood gas analysis as 8 on a 1–10 likert scale (SD 1) (Fig. 1). The free text comments reflected the high credence to the devices such as “The method is quality assured by laboratory personnel.”, “We get the same results when comparing with central [lab] analysis.” and “No deviation from later central laboratory analysis.”. In nine EDs the POCT blood gas results were deemed less reliable for electrolyte analysis due to the lack of hemolysis detection. 13 EDs point out that the “Preanalytic factors are crucial for an accurate result” and that “The majority of result deviations can be attributed to preanalytic errors”.

Regular monitoring and function checks of the blood gas analysis machines by daily automated built-in controls on the machine side and manual checks were reported by 89% of EDs (47/53). These were performed by designated ED staff daily (24/47, 51%), and weekly (9/47, 19%). In one ED central laboratory personnel checked the machine daily, in 28% (13/47) weekly.

#### Sampling and analysis

**Fig. 2:**
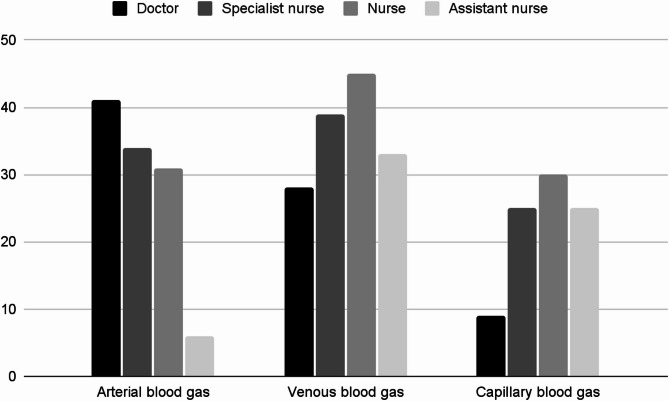
The number of EDs out of *n* = 52 with POCT blood gas analysis with formal training in blood gas sampling by occupational category. The bars represent the occupational category grouped on the x-axis by blood gas sample type and number of EDs on y-axis (n)

Most EDs (47/52, 90%; 1 non responder) provided formal training in arterial blood gas sampling to physicians. All EDs answered that nurses receive formal training for sampling venous gasses (52/52, 100%; 1 non responder). Fewer EDs provided training for capillary blood gas sampling, and primarily to nurses and assistant nurses (33/52, 64%; 28/52, 54%; 1 non responder) (Fig. 2).

Training on POCT blood gas machine handling and blood sample analysis is offered as a one time training session in most EDs, usually upon employment (20/40, 50%; 13 non responders). 30% of EDs (12/40; 13 non responders) have scheduled repeated training for all staff at least once every other year. Three EDs (3/40, 8%; 13 non responders) had no training on machine handling at all, in five EDs (5/40, 13%; 13 non responders) the training frequency was unknown.

#### Incorporation into ED workflow

Of the 33 EDs reporting monthly blood gas samples performed, nine departments reported sampling on more than 50% of total ED patients. Eight departments sampled 30–50% of their total patients, 11 departments sampled 10–30%, and five departments less than 10%. The majority of EDs facilitated an automatic transfer of POCT blood gas analysis results to the electronic health records (47/53, 89%).

44% of EDs (22/50, 44%; 3 non responders) utilize local guidelines to define indication and usage of blood gas samples in patient workup. Guidelines replacing central laboratory analyses with POCT blood gas analysis were found in 34 of 51 EDs (67%; 2 non responders). The most common analysis to be replaced was electrolytes (22/34, 65%), second and third most were glucose and hemoglobin (12/34, 35%; 9/34, 27%). Shorter time to result was the main purpose for replacing laboratory analysis with POCT (27/35, 77%; 18 non-responders). Other purposes reported were having results ready before the doctor went to see the patient, closed central laboratory night time and lower costs for analysis and transportation compared with central laboratory utilization.

83% of EDs with POCT blood gas analysis capacity (44/53; 9 non responders) responded on economic aspects of POCT. After summarizing key statements in the given answers we chose to describe three groups of economic POCT implementations. The first group of EDs (12/44, 27%) had no measures in place to record, analyze or evaluate POCT use and expenses. Usage is unrestricted. The second group of EDs (6/44, 14%) had identified problems related to unrestricted POCT usage like double sampling and higher expenses for consumables. These EDs analyze POCT usage and plan to implement guidelines to make POCT use more effective. There is an overall consensus on the cost-effectiveness of POCT in the ED when implemented correctly. The third group of EDs (26/44, 59%) had analyzed POCT use and utilize guidelines and regular economic follow ups. Double sampling has been addressed and costs for analysis, consumables and maintenance are monitored. In some EDs, certain tests, e.g. creatinine were not performed with POCT due to higher costs compared to central laboratory utilization. Sampling restrictions for certain patient categories are in place to minimize expenses, unnecessary screening and potential overdiagnosis. Training of staff prevents preanalytic errors and reduces waste of consumables.

## Discussion

In this national study covering all Swedish EDs we found that every ED utilized some kind of POCT test with the most prevalent being blood gas, Hb, glucose and CRP. Only a minority of EDs (13%) reported the use of POCT white blood cell count, whilst the majority still needed to order blood panels via central laboratory. Almost 18% of all EDs had access to POCT creatinine, mainly through blood gas analysis used almost exclusively to speed up the processing time before contrast enhanced CT.

Interestingly, only two EDs reported having POCT with troponin despite evidence to support that such testing shortens the ED length of stay by supporting transporting decision and management on route [1, 7, 20]. The American Cardiovascular Society recommends POCT for troponin when central laboratory analysis is not immediately available [21]. Central laboratories are generally available in Sweden which adopted both troponin and high sensitivity troponin early. However, large parts of Sweden are sparsely populated with small rural EDs and long ground-based transport times to tertiary care hospitals for acutely ill patients where rapid management decisions are necessary and POCT troponin could play an important role. In general, POCT utilization apart from blood gas, Hb, glucose and CRP remains low in Sweden. This is consistent with prior findings from other countries that describe the same phenomenon [22–24]. Despite evidence of potential economic gains when using POCT, the overall POCT uptake and expansion towards e.g. troponin or leukocyte analysis remains low. This may be attributed to less confidence in POCT as a method but also to a structural problem in hospital-based EDs, which is a common setup in Sweden. Here, central laboratory capacity in smaller hospitals needs to be fully utilized in order to be economically efficient, thus reducing incentives for a changeover to POCT [25]. Regardless, the variability of POCT analysis and general lack of specific analysis like troponin and leukocytes is important to understand when designing and implementing guidelines related to ED management.

Together with CRP, blood gas analysis was the most prevalent POCT accessible in 75% of EDs in Sweden, with a wide utilization range (0.3–80% of ED population, Table 1). In 17 (32%) of the EDs with high utilization (over 30% of patients), POCT blood gas analysis was triggered by, or incorporated in, standard operating procedures. This includes triage systems like WEST that require venous blood gas analysis for a considerable share of chief complaints. Hence, adoption of the WEST triage system will likely increase usage of POCT. Incorporating POCT into ED practice may improve patient safety and reduce the number of medical errors according to a consensus paper recently published, which highlights the emerging relevance of POCT in routine ED use [26].

The reliability of blood gas analysis results was generally considered high in our study with a mean estimated reliability of 8 on a scale from 1 to 10. In the following free text comments, only a minority stated systematic problems with hemolysis or other preanalytic components. Despite these concerns, the reported reliability was not negatively affected. Prior studies focused mainly on actual values ​​of deviation for e.g. potassium levels between POCT and central laboratory testing. However, they do miss out on the personnel perspective of ED staff to determine the actual relevance of these deviations [8]. Based on our findings with high reliability and trust towards POCT results, this seems to be of minor importance to staff. Whether this is due to lack of knowledge or the variability is deemed clinically irrelevant is unknown. Notably, all blood gas analysis machines are compliant with the national Swedac standard (SS-EN ISO 15189) ensuring an external maintenance process at least once a year and the majority (47/53 EDs) performed daily checks which likely provides assurance to the departments. However, it is crucial that ED staff receive regular refresher courses to ensure the analytical accuracy of the results. This is especially important as ordinary central laboratory staff rarely maintain and check on-site devices in the ED on a daily basis [4, 8]. Although all EDs trained their nurses in blood gas sample analysis, only 30% of EDs had a recurrent training schedule which indicates room for improvement.

Despite these different guidelines and workflow applications, it remains unclear if POCT blood gas analysis is a cost-effective and patient-centered method for improving patient care. An Australian study could demonstrate reduced 30-day readmission rates and lower in-hospital death rates for rural ACS patients when using POCT troponin within a structured workup [9]. Other studies stated that almost unlimited availability leads to duplicate testing, increasing costs and overdiagnosis [8, 27]. Even in our study we found almost one third of answering EDs (27%) neither restrict POCT blood gas use nor follow up on costs. On the other hand, more than half of answering EDs (59%) were aware of the costs of POCT blood gas analysis in comparison to central laboratory utilization, including indirect costs such as consumables, reduction of preanalytical errors and double sampling. This is in line with previously published systematic reviews that find evidence for POCT’s cost-effectiveness if implemented correctly [16, 28, 29].

### Limitations

We reached a response rate of 100%, including all of Sweden’s 71 hospital-based EDs, generating a solid foundation for our data. Nevertheless, the quality of answers for some questions varied within the study and non-responses limited the generalizability of results. Open text questions on e.g., evaluation and monitoring of the analytical performance or other POCT apart from blood gas analysis poses the risk that the question can be interpreted and answered differently by different EDs. We did not query about specific routines for maintenance of POCT devices, which may affect reliability, as these routines may differ between manufacturers and local conditions within the hospital. However, there was little variability in the open-ended questions, indicating no outlying device of analysis related to reliability. Furthermore, the majority of EDs (47/53) had daily maintenance on blood gas devices, which likely influenced perceived reliability more than the specifics of the routine. We did not survey the availability of analysis in the central laboratories at each site and their turnaround times which limits the analysis regarding the potential effect of increasing POCT in Swedish EDs.

## Conclusions

The majority of Swedish EDs have access to modern POCT blood gas analysis incorporating even Hb, glucose, electrolytes and to some extent creatinine analyses. Its use ranges from routine use triggered by standard operating procedures in the majority of EDs to non-routine use for only certain indications. The overall utilization of other POCT remains low apart from Hb, glucose and CRP – that despite good evidence for cost-effectiveness, reliability and accelerated patient flow for POCT.

Avoiding preanalytical errors is crucial for safe, sustainable and cost-effective POCT utilization. Our data suggests that repeated training in machine and sample handling is insufficient and needs to be addressed in the majority of Swedish EDs.

## Electronic supplementary material

Below is the link to the electronic supplementary material.

Supplementary Material 1

Supplementary Material 2

## Data Availability

The dataset used and/or analysed during the current study are available from the corresponding author on reasonable request.

## Abbreviations

ED: Emergency Department
ICU: Intensive Care Unit
IQR: Interquartile ranges
POCT: Point of care testing
SATS: South African Triage Scale
SOP: Standard operating procedure
WEST: West coast System for Triage
