# Supplementary material for: National survey on availability, use and clinical impact of point-of-care blood analysis systems in Swedish emergency departments
Source: BMC Emerg Med. 2025 Jun 7;25:93. doi: 10.1186/s12873-025-01251-7 (PMC12145587; doi:10.1186/s12873-025-01251-7)
Supplement: Supplementary file 1 — Supplementary Material 1 [file 12873_2025_1251_MOESM1_ESM.pdf]

Tillgänglighet och klinisk användning av blodgasanalyssystem på svenska akutmottagningar.

☐ Obligatoriska frågor är markerade med en stjärna (*)

1. Vilket sjukhus/akutmottagning svarar du för? *

2. Har er akutmottagning utrustning för blodgasanalys på kliniken? *

Ja

Nej

3. Vilket instrument/tillverkare för blodgasanalys har er klinik?

4. Vilken apparat/analysmetod för blodgasanalys har er klinik?

5. Hur skulle du bedöma tillförlitligheten på resultaten från blodgasanalysen?

	1	2	3	4	5	6	7	8	9	10
Ingen tilltro - Full tilltro										

6. Varför svarade du som du gjorde på förra frågan?

7. Har ni någon rutin för tidvis (daglig, veckovis etc) kontroll av era analysinstrument?

Ja

Nej

8. Om Ja, hur ser den ut?

9. Hur ofta görs en genomgång/kontroll av servicetekniker eller motsvarande personal?

10. Vilken/vilka personalkategorier har formell utbildning på er klinik för provtagning av arteriell, venös respektive kapillär blodgas?

	Läkare	Specialistsjuksköterskor	Sjuksköterskor	Undersköterskor	5
Arteriell Blodgas					
Venös Blodgas					
Kapillär Blodgas					

11. Hur ser utbildningsrutinen ut för de med behörighet för provtagning (hur ofta ges utbildningen)?

12. Vilken/vilka personalkategorier har formell utbildning på er klinik för analys av arteriell, venös respektive kapillär blodgas?

	Läkare	Specialistsjuksköterskor	Sjuksköterskor	Undersköterskor	5
Arteriell Blodgas					
Venös Blodgas					
Kapillär Blodgas					

13. Hur ser utbildningsrutinen ut för de med behörighet för analys (hur ofta ges utbildningen)?

14. Har ni PM/skriftlig rutin på när arteriell, venös respektive kapillär blodgas ska tas?

Ja

Nej

15. Om Ja, bifoga dem gärna:

16. Finns det någon rutin där blodgasanalys ersätter klassisk venös provtagning som analyseras på labb?

Ja

Nej

17. Om ja, specificera gärna vilka tester (ex. Elektrolyter, Hb, kreatinin, glukos etc).

18. Om Ja, Vad är syftet med att använda blodgasanalys istället för labb-baserad analys?

19. Har ni automatisk överföring av analyssvar till ert journalsystem?

Ja

Nej

20. Vilka ekonomiska aspekter är beaktade i rutinerna kring blodgasanalys på kliniken (ex. Begränsad provtagning pga kostnad, annat ekonomiskt ställningstagande)?

21. Hur många arteriella, venösa och kapillära blodgasanalyser görs på er klinik per månad?

22. Hur ser den procentuella fördelningen ut mellan dessa tre typer av blodgaser?

23. Vilka andra patientnära analyser utförs på er klinik?

24. Hur många patientbesök har er akutmottagning på ett år?
