# Supplementary material for: National survey on availability, use and clinical impact of point-of-care blood analysis systems in Swedish emergency departments
Source: BMC Emerg Med. 2025 Jun 7;25:93. doi: 10.1186/s12873-025-01251-7 (PMC12145587; doi:10.1186/s12873-025-01251-7)
Supplement: Supplementary file 2 — Supplementary Material 2 [file 12873_2025_1251_MOESM2_ESM.pdf]

Machine Translated by Google

Availability and clinical use of blood gas analysis systems in Swedish emergency departments.

Required questions are marked with an asterisk (*).

1. Which hospital/emergency department are you responsible for? *

2. Does your emergency department have equipment for blood gas analysis in the clinic? *

And

The best

3. Which instrument/manufacturer for blood gas analysis does your clinic have?

4. What device/analysis method for blood gas analysis does your clinic have?

Machine Translated by Google

5. How would you rate the reliability of the results from the blood gas analysis?

1 2 3 4 5 6 7 8 9 10

No trust - Full trust

6. Why did you answer the previous question the way you did?

7. Do you have a routine for periodic (daily, weekly, etc.) checking of your analysis instruments?

And

The best

8. If Yes, what does it look like?

9. How often is a review/check carried out by service technicians or equivalent personnel?

Machine Translated by Google

10. Which staff categories have formal training at your clinic for arterial, venous and capillary blood gas sampling?

	Doctors	Specialist nurses	Nurses	Assistant nurses	5
Arterial Blood Gas					
Venous Blood Gas					
Capillary Blood Gas					

11. What is the training routine for those authorized to take samples (how often is the training provided)?

12. Which staff categories have formal training at your clinic for arterial, venous and capillary blood gas analysis?

	Doctors	Specialist nurses	Nurses	Assistant nurses	5
Arterial Blood Gas					
Venous Blood Gas					
Capillary Blood Gas					

13. What is the training routine for those with analysis qualifications (how often is the training given)?

14. Do you have a memo/written routine for when arterial, venous and capillary blood gases should be taken?

Machine Translated by Google

And

The best

15. If Yes, please attach them:

16. Is there any routine where blood gas analysis replaces classic venous sampling that is analyzed in a lab?

And

The best

17. If yes, please specify which tests (e.g. electrolytes, Hb, creatinine, glucose, etc.).

18. If Yes, What is the purpose of using blood gas analysis instead of lab-based analysis?

19. Do you have automatic transfer of analysis results to your medical record system?

And

The best

Machine Translated by Google

20. What financial aspects are taken into account in the routines surrounding blood gas analysis at the clinic (e.g. limited sampling due to cost, other financial considerations)?

21. How many arterial, venous and capillary blood gas analyses are performed at your clinic per month?

22. What is the percentage distribution between these three types of blood gases?

23. What other patient-centered analyses are performed at your clinic?

24. How many patient visits does your emergency department have in a year?
